# Morbidity compression in myocardial infarction 2006 to 2015 in terms of changing rates and age at occurrence: A longitudinal study using claims data from Germany

**DOI:** 10.1371/journal.pone.0202631

**Published:** 2018-08-23

**Authors:** Siegfried Geyer, Sveja Eberhard, Bernhard Magnus W. Schmidt, Jelena Epping, Juliane Tetzlaff

**Affiliations:** 1 Medical Sociology Unit, Hannover Medical School, Hannover, Germany; 2 Local Statutory Health Insurance of Lower Saxony (AOK Niedersachsen), Hannover, Germany; 3 Clinic for Nephrology, Hannover Medical School, Hannover, Germany; Universidad Miguel Hernandez de Elche, SPAIN

## Abstract

**Background:**

According to James Fries morbidity compression is present if morbidity rates are decreasing to a larger extent than mortality rates. Compression also occurs if age at onset is increasing at a faster pace than age at death. These two variants of the compression hypothesis were formulated as a population concept. Compression has seldom been studied with a specific disease as application.

**Methods:**

Morbidity compression was examined in terms of myocardial infarction (MI) by using German claims data covering the years 2006 to 2015. The findings are based on an annual case number of about 2 m women and men aged 18 years and older. Analyses were performed by means of proportional hazards regression and by using linear regression.

**Results:**

Decreases of morbidity rates were more pronounced than those of mortality. For men, the hazard ratio for contracting MI in 2015 as compared to 2006 was hr = 0.66 and hr = 0.71 for the female population. The respective results for mortality were hr = 0.75 in men and hr = 1.0 in women. They can be interpreted in favor of morbidity compression. For the subgroup of women and men with MI, changes of onset age revealed marked gender differences. For 2015 as compared with 2006, age at MI-occurrence in men increased by 10.5 months as compared to an increase of 10.4 months for age at death. In women changes were smaller and statistically not significant. The findings referring to women have to be interpreted against the backdrop of higher onset age and higher age at death than in men.

**Conclusions:**

Taken together, morbidity compression has occurred in terms of decreasing MI-rates as well as in terms of increased onset age in men. It can be concluded that both processes have led to an improvement of healthy lifetime. Decreasing morbidity rates in women are also pointing towards morbidity compression, a finding that is not complemented by changes of onset age. Our data are demonstrating that morbidity rates and age at onset may vary independently. From this perspective morbidity compression is a multi-faceted phenomenon.

## Introduction

In the 1980s James Fries formulated an optimistic perspective on the development of population health [1]. His hypothesis of morbidity compression states that prevention, improved living conditions and socio-economic factors are contributing to a prolongation and gains of healthy lifetime [2–5]. He assumed morbidity compression not only to take place in higher age groups, but also in earlier periods of life as myocardial infarctions and states of minimal morbidity may already occur around the age of 50 [6] (p.1) [3](p.164). Over the years Fries published several papers on morbidity compression that have contributed to further refinements of the concept, but they are also giving rise to the need of clarification. At first it has to be emphasized that morbidity compression refers to relationships between morbidity and mortality, but for compression to occur it is not necessary that life expectancy or mean age at death are changing [3, 6]. From Fries’ writings morbidity compression may be conceptualized in two ways. It has to be emphasized that they may not necessarily occur jointly, but also independently.

1. The first formulation refers to morbidity compression as the relationship between decreasing morbidity and mortality rates [7](p.210). Compression is present if age-specific morbidity rates are decreasing more rapidly than age-specific mortality rates [2] (p.811). By analyzing rates, populations have to be considered over defined observation periods.
2. According to the second conceptualization compression occurs “…if the age at first appearance of aging manifestations and chronic disease symptoms can increase more rapidly than life expectancy” [2] (p.810) [8](p.1638). Empirically this has to be examined by analyzing changes of onset age in relation with life expectancy or age at death. With respect to the analyses below it has to be noted that this refers only to the subset of a population with a myocardial infarction or to those who are dying.

In his early papers Fries was referring to life expectancy in terms of a maximum biological lifespan [1, 2, 9], a term that has aroused much controversy among demographers [10, 11]. For empirical work the assumption of a fixed lifespan leads to study designs that may confine analyses to morbidity without having to collect information on mortality from the same dataset. If the assumption of a maximum lifespan is abandoned or left as unknown, morbidity and mortality have to be considered together, but in empirical studies this had not always been done.

Irrespective of the need for some conceptual clarifications, Fries hypothesis has stimulated many empirical studies. They are covering a broad variety of outcomes, ranging from physical diseases, mental decay [12–14] and functional impairments to the development of health care costs [4, 15], postponement of retirement age [9], or the development of self-determined living in old age [16]. The studies published so far can be divided into work on general health/ impairments of everyday activities, on mental impairments, and on specific diseases [17].

The largest number of studies including Fries’ own work deals with general health and impairments of everyday activities. His study on runners examined relationships between physical activity and longevity [6, 8]. From 1984 on physically active women and men were compared with less active controls. In 2005 health impairments and the utilization of health services were assessed. In the active group the prevalence of health impairments was lower, and also the risk of death. These findings were confirmed in a later study where health was conceptualized as a count of diseases and impairments [18]. Romeu used data of the Health and Retirement Study for examining changes of everyday impairments [19]. After having adjusted for age, cohorts surveyed later had lower degrees of everyday impairments than cohorts surveyed earlier, permitting the conclusion that compression had taken place. Manton [20] combined data of six surveys conducted between 1982 and 2004 by considering respondents aged 65 years and older. Self-care limitations impairments of everyday activities were used as outcomes. In women and in men the later surveyed cohorts were living longer with lower degrees of impairments than earlier ones, and cohort effects were most pronounced at the upper end of life, findings that can be interpreted as morbidity compression. This was confirmed in a study based on Medicare-based claims data [21]. Graham et al [22] used data from New Zealand from 1981 to 1996. They reported increasing rates of functional limitations, but this occurred in terms of moderate degrees, while the number with more severe impairments remained at the same level. These findings cannot be interpreted as compression, but rather as morbidity expansion [23].

A German study with routine data examined the long-term development of the need of care by considering amount of need and geographical region [24]. Although regional differences emerged, the general trend went towards a general increase of need of care, but severe morbidity decreased, thus rather pointing towards a dynamic equilibrium. This was at least partly confirmed by a second German study on the same outcome [25]. The findings of another German study with claims data was pointing into the same direction as multimorbidity rates were increasing from 2005 to 2014 [26].

This overview of research with general health measures as outcomes was intended as representative, but not as exhausting. Studies with subjective health measures, functional impairments and disability are representing the bulk of literature on morbidity compression. Beltran-Sanchez considered this as a severe shortcoming of the current state of research, and he pointed out that specific diseases should be considered as endpoints [27].

Cognitive impairments are ranging between subjectively assessed health and specific diseases [28]. A study conducted between 1993 and 2002 reported that in the first wave the proportion of impaired individuals aged 70 years and older was 12.2%, while at the second wave it dropped to 8.7% [13]. These findings were confirmed in a second study with women and men aged 65 year and older [29]. In all of these cases better cognitive functioning was associated with higher longevity. A study on dementia with claims data reported decreasing incidences between 2006/2007 and 2009/2010 and dementia-free lifetime was increasing. The authors concluded that morbidity compression had occurred [12, 29].

A US-based study considered morbidity changes in terms of physical diseases in samples surveyed between 1998 and 2004 and 2004 and 2010 [30]. It turned out that the more recently surveyed cohorts had higher rates of cancer, diabetes, lung disease and high blood pressure as compared to subjects of the same age group from the earlier waves. These findings are contradicting the compression hypothesis as they were pointing towards higher than lower degrees of morbidity. A German study on diabetes type 2 reported stable incidence rates for 2005 to 2013 in middle and higher age groups, but rates in the 18 to 39-year olds were increasing and age at occurrence was shifting downwardly [31, 32]. So far the development may be described as morbidity expansion, but further analyses will be necessary to differentiate between expansion and a dynamic equilibrium [33], i.e. that patients with diabetes may live longer and with better quality of life than in earlier times. In another study covering 2008 to 2014 different types of stroke (cerebral infarction and haemorrhagic stroke) were examined [34]. While no changes over time occurred for cerebral infarction, the rates of haemorrhagic stroke decreased, thus morbidity compression had occurred only in a subtype making up only 20% of all onsets.

Myocardial infarction (MI) is one of the most frequently occurring diseases, and first incidence as well as case-fatality rates were decreasing since the 1970s [35]. Incidences of cardiovascular diseases were reported to having declined between 1970 and 2000, accounting for about 60% of the increasing life expectancy in the USA [36]. Another US-based study used Medicare-based data of 18,670 women treated between 1999 and 2009 [37]. Besides a general decrease of cardiovascular risks, mean age at onset increased while survival rates remained unchanged. A US-based regional study used data of the years 1995 to 2012. A total number of 5258 myocardial infarction cases were reported, and incidences declined by 3.3% per year [38]. Another study reported decreasing rates of cardiovascular mortality in Germany where rates in males were reported to having declined since 1981, in women the same development was taking place since 1985 [39]. The “Early Indicators”-project was based on records of male US-military personnel what made it possible to observe health-related developments at population level over a period of more than 100 years [40]. Mean age at onset increased by 10 years while the gain of life expectancy at the age of 50 was only 6.6 years, indicating absolute compression of morbidity. Although morbidity compression was found, the age groups where it occurred were not reported.

Fries assumed that age at death would be determined by a biologically limited life expectancy. Although this assumption is reasonable, numeric estimations of life expectancy have always become outdated. There is evidence that since 1840 the highest measured annual life expectancy had increased by about three months per year [41]. In recent decades this was due to changes in the higher age groups. This holds for the USA [42], for Germany [39], and for several European countries [43, 44], therefore developments of morbidity have to be considered alongside developments of mortality.

Taken together, the findings on morbidity compression appears as heterogeneous. After having reviewed a large number of studies, Crimmins and Beltran-Sanchez concluded that there was evidence in favor of compression as well as counterevidence [42]. It has however to be emphasized that the findings have to be interpreted against the backdrop of the outcomes chosen, the certitude onsets can be dated with, by the time period considered and by the country where the data were collected. Gender differences have also to be taken into account.

In the following analyses morbidity compression will be examined for the case of myocardial infarction (MI). This outcome was chosen because it is frequent, it can be diagnosed and dated with sufficient accuracy, and studies on morbidity compression on MI are rare. Against the backdrop of the considerations above the following topics will be dealt with:

- It will be examined whether MI-rates were decreasing over the observation period and whether MI-rates were decreasing to the same extent or stronger than mortality rates. This refers to the abovementioned first formulation of morbidity compression as decreasing rates of myocardial infarction in connection with age-standardized mortality rates.
- It will be examined whether age of onset and age at death have shifted upwardly over the observation period. This refers to the second formulation of morbidity compression as change of mean age at MI-onset as related to changes of mean age at death.
- Does morbidity compression in terms of MI occur in specific age periods or does it take place over the whole age range? This third line of analysis refers to Fries’ considerations that morbidity compression may not only take place at the end of life but over the whole age range. It refers to morbidity only, but it integrates the considerations on changes of age and changes of rates.

## Materials and methods

### Database

The data used for the following analyses are pseudonymised claims data of a German statutory health insurance, the AOK Niedersachsen (AOKN). The database is covering the years 2005 to 2015 with about 2 m insured per year aged 18 years and older. It does not depict a sample, but a complete population. Power analyses were performed for Cox-regression. Setting the probability of an endpoint event to 0.01, the significance level to p = 0.01, the power of testing to 0.8, and the effect size in terms of hazard ratio to hr = 0.1, the necessary case number is N = 882. This was exceeded in all lines of analysis. Comparative analyses have shown that the distributions of age and gender of our insurance population, those of Lower Saxony and of Germany did not differ, but the insurance population had a higher proportion of individuals with lower occupational qualifications [45]. This implies that health and life expectancy of our population should be lower than at nationwide level.

All residents of Germany must have health insurance, and in 2011 only 0.2% were uninsured [46]. Below a certain income threshold insurance with the statutory system is mandatory, and in 2011 this applied to 89% of all permanent residents. Insurance premiums within the statutory system are fixed to 14.6% of the pre-tax income. Spouses without employment and children are insured free of charge, irrespective of family size. Health care providers are not paid by patients but by health insurances, thus separating doctor-patient relationship from financial issues. Within the statutory health care system, the amount of health care coverage is the same for all insured individuals. Regular adaptations of coverage are carried out according to the development of medical treatment. The private health insurance sector covers state- and self-employed individuals and those above a certain income threshold (11% of all residents). Insurance premiums are calculated at an individual basis according to predefined health risks [47].

Claims data from statutory health insurances are fairly complete as all shifts of money from insurers to providers are registered. Supplementary payments are rare, at least those falling within the topic of this paper. Health insurance records are including socio-demographic information as well as data on unemployment, education, income, occupation, in- and outpatient treatment and medications with the respective dates of occurrence. This time-related structure makes it possible to establish event sequences. A further advantage of claims data is the absence of dropouts. Staying in a hospital or living in an institution (e.g. a retirement home or a prison) does not lead to exclusion from analysis. Diseases and deaths are recorded within the same dataset thus making it possible to analyze them in context. The data were systematically checked for errors, consistency, duplicates, and for the correctness of the temporal order of events.

The following variables will be used:

Classifications of **myocardial infarctions** (MI) are based on hospital diagnoses and coded according to ICD10. Cases were classified as myocardial infarctions if one of the following diagnoses were assigned: ICD-10: I21.0 to I21.9 (acute myocardial infarction with the fourth digit denoting the location) of the International Classification of Diseases (ICD) as issued by the World Health Organization (http://www.who.int/classifications/icd/en/). In case of several events only the first one in a chronological order was counted. Cases of recurrent myocardial infarctions (ICD-10: I22) were not considered. Nevertheless, it cannot be excluded that recorded I21.X-cases were falsely classified as first events. In order to reduce the likelihood of misclassifications, a pre-observation period of one year was introduced. It was counted from the beginning of the observation period on, and all MIs occurring within this period were excluded, thus leading to a shortening of the total observation time. The information base for defining pre-observation periods for MIs is scarce as not many studies are available, and the figures are varying according to health care systems as they are setting up the framework of data collections from different countries. Published studies are consistent that the majority of recurrences occurs within 12 months after first MI, that the likelihood of an event is increasing with the age of patients [48, 49], and that recurrence risks were decreasing in recent years [49]. In a study from the US it was reported that 14% of the women and 13.5% of men had a recurrence within 12 months after first MI [50], and in a UK-based study 5.6% of men and 7.2% of women were reported to having had a second MI within the same period [51].

**Mortality** has to be included as the second indicator determining morbidity compression. In the health insurance data death is recorded with its precise date as it terminates health insurance membership.

**Calendar year** is the main variable for stratification if morbidity compression is examined. The insured can be located with respect to their terms of insurance, and every event can be also be located by its date.

#### Insurance status

The insurance population is divided into employed, family insured (family members insured free of charge), pensioners, and unemployed as morbidity and mortality risks are differing over these groups. The analyses to follow will focus on changes of morbidity and mortality over calendar years. Insurance status has to be controlled for because the structure of the insurance population may change over time. Individuals without employment and those officially registered as unemployed were shown to having higher health risks than those who were employed [52, 53]. Ignoring insurance structure would lead to erroneous conclusions. This also refers to increasing labour force participation of women over time and to changes of the age at retirement.

Age had to be introduced as a control variable as both outcomes are age-dependent. For MI and for death, age at event occurrence was used, and for censorized cases age at the end of observation was used.

### Analyses

According to the three topics formulated at the end of the introduction, analyses are performed in separate lines of analysis. To date no statistical procedures are available that are permitting to examine the three different aspects of morbidity compression simultaneously. At the **first step** changes of MI- and mortality rates over time are examined by using Cox-proportional hazards model for calculating hazard ratios for MI and for death. The Cox-model is based on the occurrence/ non-occurrence of events, i.e. the dependent variable is scaled in categories. In analyses of morbidity compression calendar year is the main variable of interest. Using it leads to different survival curves, one for every year with one (in the present case the first year of observation) as the standard of comparison. Age at occurrence of an event has to be included as the risks of MI-onset and of death are increasing with age. Furthermore, beginning and end of insurance periods are defining the lengths of observation periods. They have to be included because events can only be observed in these intervals, thus the likelihood of observed occurrence is dependent on the length of observation periods. As MI-onsets are first events and death can occur only once, censoring is effective as right-censoring, i.e. it refers to events occurring after the end of observation.

At the **second step** morbidity compression will be considered in terms of changes of MI-onset age, and age at death. While the occurrence of myocardial infarctions or deaths can be analyzed by means of survival models, changes of age at onset or at death are more difficult to examine. They have nevertheless to be considered as the postponement of onset age over time was formulated as the second variant of morbidity compression [2]. Calendar year was the most important independent variable, and type of insurance had to be controlled for.

By searching appropriate methods, estimation problems were encountered. Analyses of changes of age at occurrence are considering only cases with an event of interest (onset or death), all other cases are excluded. If only a subset of subjects is considered, sample selection bias may occur, because this subset may not be representative of the whole population. A model addressing this problem was proposed by Heckman [54], also known as Tobit-II- model. It is treating individuals without a defined event as censorized cases, and occurrence of events (categorical scale) and their dates (metric scale) are included in a single equation model by using maximum likelihood estimation. Normal distribution of errors and homogeneous variances (homoscedasticity) are required for obtaining unbiased estimates. While heteroscedasticity may be amended by using bootstrapping, the distribution of censorized cases are causing serious problems that cannot be resolved. In the years prior to the end of observation (i.e. between 2006 and 2014) censorizations were caused by leaving the insurance population. In the last year (2015) a different censoring mechanism was effective, because the observation period ended for all subjects, i.e. it was caused arbitrarily the availability of data. Different types of censorizations are causing estimation problems for the Heckman-model, thus making it unsuitable for tackling our research question. The numbers of healthy life years are often estimated using the Sullivan-Method [55] which is based on the analysis of life tables. For our purposes this approach has disadvantages that led us to abandon it. As the Sullivan-Method is based on tables depicting populations by aggregated data, changing population structures cannot be taken into account. A way out might be to create tables for subpopulations thus leading to a large number of tables that have to be compared. As a second reason, the Sullivan- Method is extrapolating trends what is appropriate if some data are missing or if only aggregated data are available. In contrast, our study requires that the findings are controlled for population structure, and the data are available at micro-level.

Preparatory analyses led to the following decisions for examining the second part of the compression hypothesis.

- Finally, it was decided to estimate changes of event occurrence (myocardial infarction and death) by means of Ordinary Least Squares R egression (OLS). “Calendar year” and “type of insurance” were used as independent variables and date of occurrence was used as dependent variable. Comparative analyses have shown that the substantive conclusions concerning effects of calendar year on age at event did not differ substantially between the OLS-solution and the Heckman-model with the year 2015 being an exception as explained above. As OLS-estimates may be flawed by heteroskedasticity, the Cook-Weisberg-test [56] was performed with our prediction model. The findings were indicating a significant deviation from homoskedasticity (chi^2^(13) = 693.6; p<0.001), and further analyses led to the conclusion that this was due to “type of insurance”, in particular to the heterogeneous group of unclassified subjects. Analyses performed only with the “retired” insured as group with the highest MI-rates did not lead to different conclusions from analyses with the whole study population. Finally, it was decided to perform the analyses as reported below, and confidence intervals were based on 1000 bootstrap-samples. Bootstraps are performed by drawing samples with replacement in order to estimate statistical parameters, in the present case confidence intervals for making sure that significance tests based on normality assumptions can be applied. This technique is appropriate if distributional properties of certain parameters are unknown, if they deviate from normality, or if the underlying population is not known so that the study population is used for making inferences [57].
- Against the backdrop of demographic aging, the proportion of elderly insured will increase over time. This will lead to a clustering of elderly people and to an increasing number of myocardial infarctions. OLS- regression will then lead to an overestimation of increases of age at onset without risks of MI incidence having changed over time. In order to avoid biased estimates, a sampling procedure had to be applied: For every age stratum the calendar year with the lowest number of cases was sought, and then random samples for all age groups were drawn for every calendar year in order to obtain equal case numbers for every year of age. Then the OLS-regressions were performed with the resulting dataset. For the case of population ageing, regression analyses with the sampling solution should yield more conservative estimates than analyses with the complete study population. Comparisons of the two approaches revealed that this was indeed the case. As it will be shown below, the corresponding effects in women turned out as inconsistent and not statistically significant, irrespective of the approach chosen.
- The OLS- model at the second step of analysis includes only cases with myocardial infarction or deceased individuals. Age at occurrence is used as dependent variable with months as unit of measurement. In all analyses the structure of the insurance population has to be controlled for in order to rule out effects of changing compositions of the population insurance structure over time. For morbidity compression to be present, age at occurrence of events has to move upwardly as time (depicted as calendar years) proceeds. It is entering analysis with the first year of observation as reference category. In the regression model the reference category is depicted as intercept at the y-axis (scaled in months), and changes (i.e. unstandardized regression effects) are appearing as intercept shifts between the reference category (first year of observation) and the subsequent ones.
  This is expressed by the following equation system:
  $${CIM}_{1..9}=\beta_{0}+\delta_{1..9}\;{YR}_{2007..2015}+\gamma_{1}\;{Pop}_{2}+\gamma_{2}{Pop}_{3}+\gamma_{3}{Pop}_{4}+\gamma_{4}\;{Pop}_{5}+\varepsilon$$
  “CIM” corresponds to changes in months as compared to the first year of observation as reference category; β_0_ denotes the intercept that in the present case equals the first year of observation (= 2006); δ_1_..9 YR 2007..2015 denote the effects for year 1 (= 2007) to year 9 (= 2015). Effects of insurance status as control variable are denoted as γ_,_ where the subscript “1” denotes the effect of the family insured (Pop2), “2” denotes the effect of pensioners (Pop3), “3” denotes the effect of the unemployed insured (Pop4), and “4” denotes the effect of unclassified insured (Pop5), and “ε” denotes the error term.

At the **third step** survival analyses are performed for examining changes of MI-onset rates by means of Kaplan-Meyer survival curves. The MI-rates of two cohorts of the same age are compared over a time period of five years, i.e. men at the age of 60 in 2006 are observed from 2006 to 2010, and those who are 60 years old in 2011 are observed over the period 2011 to 2015. The analyses are performed stratified by gender and by age for age groups 60–64, 65–69, 70–74, 75–79, 80–84, and 85–89 years. The survival curves have to be interpreted in the way that each graph displays the remaining proportion of individuals who had not had a MI until the end of the observation period. Each pair of survival curves will be tested for differences by using the log-rank test and by assuming an error probability of 5%. All analyses were performed with STATA 14 SE [58].

## Results

The basic frequencies of the relevant variables are displayed in Tables 1 and 2. It has to be noted that the adjusted mean age at MI-onset was 66.5 (Sd = 13.3) years in men and 75.8 (Sd = 13.3) years in women. The MI- rates in women were smaller than in men, and mean age at death in men was 73.0 (Sd = 13.5) years, and 81.4 (Sd = 11.9) years in women.

**Table 1:** Distribution of the variables used of the complete male population and for sample-based analyses (age at MI-onset and age at death).

		**2006**	**2007**	**2008**	**2009**	**2010**	**2011**	**2012**	**2013**	**2014**	**2015**
	**All subjects**										
**Total**		849,204	846,331	835,807	833,703	840,839	855,121	864,994	871,625	875,217	884,094
Myocardial infarction	Frequency	3492	3610	3621	3507	3487	3524	3615	3565	3511	3413
	%	0.41%	0.43%	0.43%	0.42%	0.41%	0.41%	0.42%	0.41%	0.40%	0.39%
Deaths	Frequency	14,234	14,145	14,410	14,506	14,335	14,326	14,523	15,109	14,714	15,178
	%	1.68%	1.67%	1.72%	1.74%	1.70%	1.68%	1.68%	1.73%	1.68%	1.72%
Insurance	Employed	432,778/ 51.0%	442,950/ 52.3%	443,047/ 53.0%	436,608/ 52.4%	449,713/ 53.5%	473,815/ 55.4%	486,688/ 56.3%	492,674/ 56.5%	498,859/ 57.0%	509,568/ 57.6%
status	Family insured	23,120/ 2.7%	22,284/ 2.6%	21,292/ 2.6%	21,846/ 2.6%	21,323/ 2.5%	20,195/ 2.4%	19,884/ 2.3%	19,831/ 2.3%	19,604/ 2.2%	18,985/ 2.2%
N / %	Pensioners	250,419/ 29.5%	246,765/ 29.2%	243,412/ 29.1%	239,436/ 28.7%	235,791/ 28.0%	233,656/ 27.3%	232,239/ 26.9%	229,522/ 26.3%	226,606/ 25.9%	225,569/ 25.5%
	Unemployed	96,381/ 11.4%	87,875/ 10.4%	81,496/ 9.8%	86,474/ 10.4%	84,213/ 10.0%	77,722/ 9.1%	74,055/ 8.6%	75,799/ 8.7%	75,393/ 8.6%	74,317/ 8.4%
	Others	46,506/ 5.5%	46,457/ 5.5%	46,560/ 5.6%	46,339/ 5.9%	49,799/ 5.9%	49,733/ 5.8%	52,128/ 6.0%	53,799/ 6.2%	54,755/ 6.3%	55,655/ 6.3%
**Sample**	**N = 780,820**	**2006**	**2007**	**2008**	**2009**	**2010**	**2011**	**2012**	**2013**	**2014**	**2015**
Myocardial infarction	Frequency	2867	3013	3012	2867	2816	2825	2844	2787	2735	2627
	%	0.37	0.39	0.38	0.37	0.36	0.36	0.36	0.36	0.35	0.34
Deaths	Frequency	11,843	11,655	11,696	11,687	11,263	11,044	11,061	11,195	10,810	10,875
	%	1.52	1.49	12.1	1,50	1.45	1.42	1.43	1.45	1.41	1.42

**Table 2:** Distribution of the variables used of the complete female population (survival analyses) and for sample-based analyses (age at MI-onset and age at death).

** **	** **	**2006**	**2007**	**2008**	**2009**	**2010**	**2011**	**2012**	**2013**	**2014**	**2015**
	**All subjects**										
**Total**		989,715	980,943	964,098	955,329	956,666	966,172	971,232	970,405	966,707	969,760
Myocardial infarction	Frequency	2635	2618	2542	2459	2405	246	2513	2324	2250	2167
	%	0.27%	0.27%	0.26%	0.26%	0.25%	0.26%	0.26%	0.24%	0.23%	0.22%
Deaths	Frequency	18,257	18,341	18,615	18,467	18,394	17,581	17,930	18,455	17,382	18,435
	%	1.87%	1.87%	1.93%	1.93%	1.92%	1.82%	1.85%	1.90%	1.80%	1.90%
Insurance	Employed	297,804/ 30.1%	304,628/ 31.1%	306,686/ 31.8%	309,307/ 32.4%	321,084/ 33.6%	341,922/ 35.4%	355,312/ 36.6%	362,177/ 37.3%	369,968/ 38.3%	385,182/ 39.7%
status	Family insured	180,009/ 18.2%	173,337/ 17.7%	165,649/ 17.2%	159,611/ 16.7%	155,777/ 16.3%	150,947/ 15.6%	146,605/ 15.0%	141,690/ 14.6%	137,047/ 14.2%	128,933/ 13.3%
N / %	Pensioners	393,495/ 39.8%	386,567/ 39.4%	378,927/ 39.3%	371,475/ 38.9%	364,998/ 38.2%	360,103/ 37.3%	365,895/ 36.8%	350,413/ 36.1%	343,458/ 35.5%	339,945/ 35.1%
	Unemployed	71,099/ 7.2%	68,996/ 7.0%	66,480/ 6.9%	67,533/ 7.1%	67,173/ 7.0%	65,194/ 6.8%	63,423/ 6.5%	64,875/ 6.7%	64,618/ 6.7%	63,797/ 6.6%
	Others	47,308/ 4.8%	47,415/ 4.8%	46,356/ 4.8%	47,403/ 5.0%	47,634/ 5.0%	48,006/ 5.0%	49,997/ 5.2%	51,250/ 5.3%	51,616/ 5.3%	51,903/ 5.4%
**Sample**	**N = 876,800**	**2006**	**2007**	**2008**	**2009**	**2010**	**2011**	**2012**	**2013**	**2014**	**2015**
**Total**											
Myocardial infarction	Frequency	2133	2153	2093	2040	1956	2056	2086	1926	1845	1764
	%	0.24	0.25	0.24	0.24	0.22	0.23	0.24	0.22	0.21	0.20
Deaths	Frequency	14,646	14,409	14,535	14,657	14,454	13,706	13,921	14,234	13,359	14,049
	%	1.67	1.64	1.66	1.67	1.65	1.57	1.60	1.64	1.54	1.63

### Development of morbidity and mortality rates

In *men*, the hazard ratios of MI-onsets of the years following 2006 were decreasing constantly (Table 3). The differences to the year of reference were statistically significant from 2009 on (hr = 0.83). In 2015 MI-onsets were 34% lower than in 2006 (hr = 0.66). Hazard ratios of mortality were also decreasing over the 10 years, finally reaching hr = 0.75. A similar development emerged in *women*. The hazard ratios of MI-onset were decreasing over the 10 years and from 2011 on differences between calendar years were statistically significant. MI-onset rates for 2015 were 29% lower than in 2006. Different from death and MI-rates in men and from MI in women, hazard ratios of death did not change significantly over the observation period. It has to be kept in mind that the mean age of MI-onset and at death of women was higher than of men. As morbidity rates in men were decreasing at a faster pace than those of mortality and due to stable mortality rates in women it can be concluded that compression of morbidity has occurred.

**Table 3:** Onsets of myocardial infarctions and mortality in women and men by controlling for insurance group: Hazard ratios, standard errors and confidence intervals.

		**Men: Myocardial infarction**			**Men: Mortality**		
	Year	Hazard ratio	p	95% CI	Hazard ratio	p	95% CI
Men	**2006**	Ref.	-	-	1	-	-
	**2007**	1.00	0.99	0.87–1.15	0.92	0.07	0.85–1.00
	**2008**	0.97	0.67	0.84–1.12	0.85	<0.01	0.78–0.93
	**2009**	0.83	0.01	0.72–0.96	0.90	0.02	0.83–0.99
	**2010**	0.82	0.01	0.71–0.94	0.88	<0.01	0.80–0.96
	**2011**	0.81	<0.01	0.70–0.93	0.87	<0.01	0.79–0.95
	**2012**	0.75	<0.001	0.65–0.86	0.78	<0.01	0.71–0.85
	**2013**	0.74	<0.001	0.64–0.85	0.82	<0.01	0.75–0.89
	**2014**	0.67	<0.001	0.58–0.78	0.73	<0.01	0.67–0.80
	**2015**	0.66	<0.001	0.57–0.77	0.75	<0.01	0.69–0.80
	Age (years)	1.0539	<0.001	1.0528–1.0550	1.072	<0.001	1.072–1.073
		**Women: Myocardial infarction**			**Women: Mortality**		
	**Year**	Hazard ratio	p	95% CI	Hazard ratio	p	95% CI
**Women**	**2006**	Ref.	-	-	Ref.	-	-
	**2007**	1.00	0.96	0.80–1.26	0.96	0.46	0.88–1.06
	**2008**	0.86	0.20	0.69–1.08	0.96	0.51	0.88–1.07
	**2009**	0.93	0.52	0.74–1.16	1.10	0.11	0.98–1.19
	**2010**	0.83	0.11	0.66–1.04	1.12	0.02	1.02–1.23
	**2011**	0.80	0.05	0.64–1.00	0.94	0.22	0.85–1.03
	**2012**	0.80	0.04	0.64–0.99	0.93	0.14	0.84–1.02
	**2013**	0.66	<0.01	0.53–0.83	1.02	0.64	0.92–1.12
	**2014**	0.75	0.01	0.60–0.95	1.03	0.53	0.94–1.15
	**2015**	0.71	0.01	0.57–0.90	1.00	0.88	0.91–1.11
	Age (years)	1.0593	<0.001	1.057–1.060	1.068	<0.001	1.1062–1.1073

### Changes of age at onset and age at death

In ***men*** age at onset of MI was increasing over the observation period (Table 4). Although the general trend was towards postponement of onset, the development was not completely steady. In 2015 the mean age at MI-onset was 10.5 months higher than in 2006 with a maximum of 13 months in 2014. The development of mortality was following the same pattern, but the changes occurred at a lower level. Taken together it can be concluded that in men morbidity compression has occurred, and in both cases event age was increasing, and changes in terms of onset age were higher than those of mortalit**y.**

**Table 4:** Changes of age at onset of myocardial infarction and at death in months in women and in men in terms of months: Effect sizes and confidence intervals based on 1000 bootstrap samples.

**Men**						
	**Myocardial infarction**			**Death**		
	B	p	95% CI	B	P	95% CI
**2006**	Ref.	-	-	Ref.	-	.
**2007**	3.8	0.16	-1.5–9.2	-2.3	0.16	-5.6–0.9
**2008**	5.1	0.06	-0.3–10.5	-0.2	0.88	-3.5–3.0
**2009**	5.7	0.04	0.3–11.1	0.7	0.69	-2.6–3.9
**2010**	6.6	0.02	1.2–12.1	3.0	0.08	-0.3–6.2
**2011**	9.7	<0.01	4.2–15.1	4.4	<0.01	1.1–7.7
**2012**	8.3	<0.01	2.8–13.7	6.6	<0.01	3.3–9.9
**2013**	12.1	<0.01	6.7–17.6	7.5	<0.01	4.3–10.8
**2014**	13.4	<0.01	7.9–18.9	10.5	<0.01	7.2–13.8
**2015**	10.5	<0.01	5.0–16.1	10.4	<0.01	7.1–13.7
Constant	625.2	<0.01	620.8–629.6	624.8	<0.01	621.3–628.3
**Women**						
	**Myocardial infarction**			**Death**		
	B	p	95% CI	B	p	95% CI
**2006**	Ref.	-	-	Ref.	-	-
**2007**	2.6	0.42	-3.8–8.9	2.2	0.15	-4.3–1.3
**2008**	4.3	0.18	-2.0–10.7	3.3	0.12	-3.3–2.4
**2009**	8.9	<0.01	2.5–15.3	1.6	0.02	-1.3–4.1
**2010**	1.8	0.58	-4.7–8.3	2.3	0.24	-3.6–1.8
**2011**	4.2	0.20	2.2–10.6	2.4	0.10	-3.4–2.4
**2012**	3.3	0.30	-3.0–9.7	3.3	0.09	-2.8–2.9
**2013**	5.3	0.11	-1.2–11.8	3.1	0.02	-0.9–4.6
**2014**	-1.9	0.55	-8.6–4.6	2.0	0.15	-2.7–2.8
**2015**	0.8	0.81	-5.8–7.5	4.8	<0.01	-3.1–2.5
Constant	631.7	<0.01	624.4–638.9	614.9	<0.01	609.3–619.3

**In *women*** changes of onset age and of death were less steady than in men and smaller. Also, age at death turned out as rather stable over time as the variation was always distributed around the reference year. Thus, in contrast to men, decreasing MI-rates in women were not complemented by rising age at onset and at death.

### Morbidity changes by age, period, and gender

For sake of brevity only the survival functions of the ages at origin 70–74, 75–79, and 80–84 years are displayed graphically.

**Fig 1:**
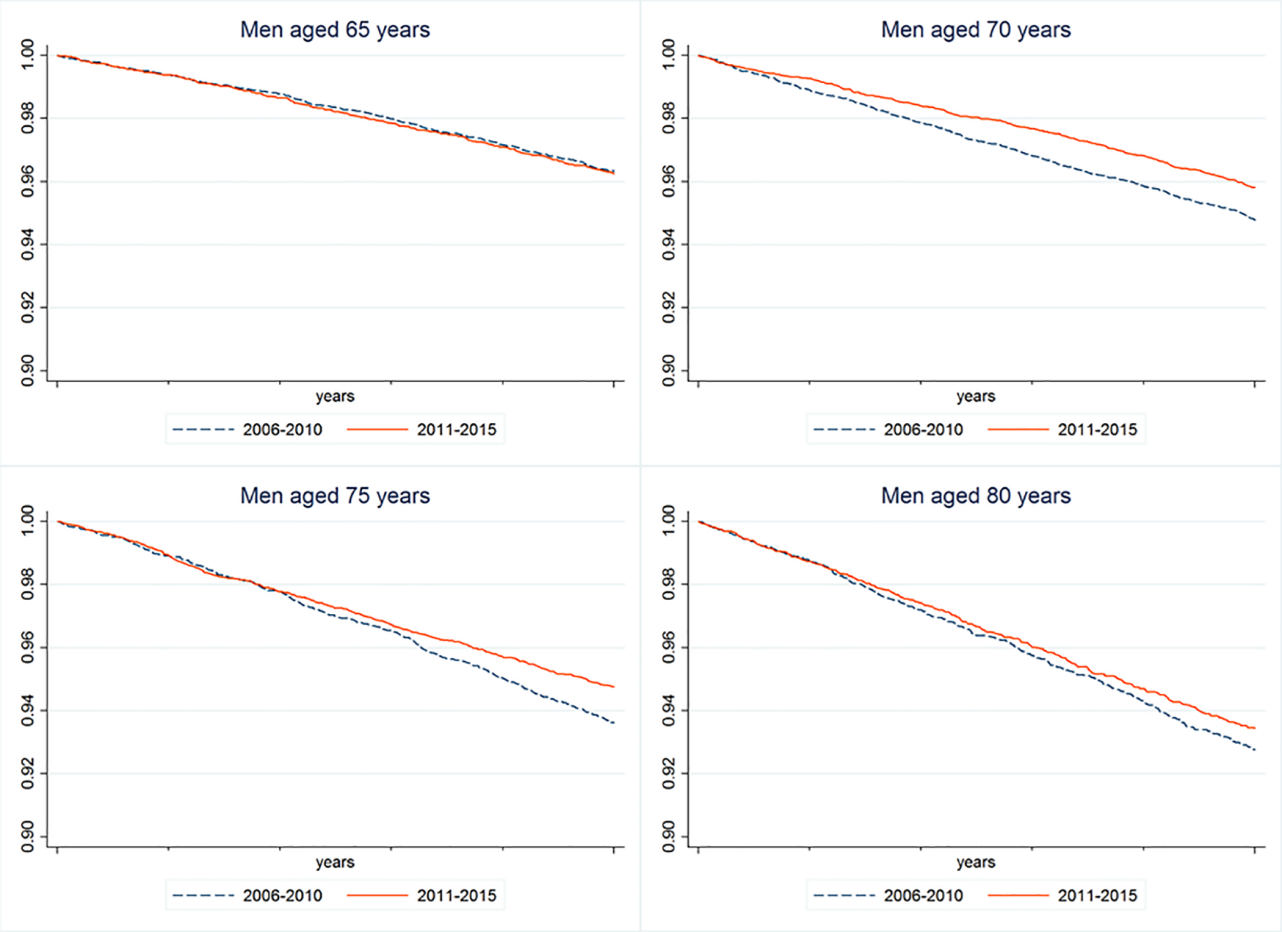
Survival curves of MI-onsets in men of different age groups (65–69 yrs., 70–74 yrs., 75–79 and 80–84 yrs.) over two periods (2006–2010 and 2011–2015).

In ***men*** (Fig 1) aged 60–64 years the survival curves of the two observation periods were statistically different (chi^2^ = 5.94; p = 0.02). For the following age segment (65 to 69 years) no statistically significant differences between time periods emerged (chi^2^ = 0.02; p = 0.88). For the age group 70 to 74 years the survival curves were differing again, indicating decreasing MI-rates over the observation period (chi^2^ = 14.98; p<0.001). The same held in the subsequent age interval of 75 to 79 years (chi^2^ = 9.08; p<0.01), but not in the last intervals considered (80 to 84 and 85 to 89 years).

**Fig 2:**
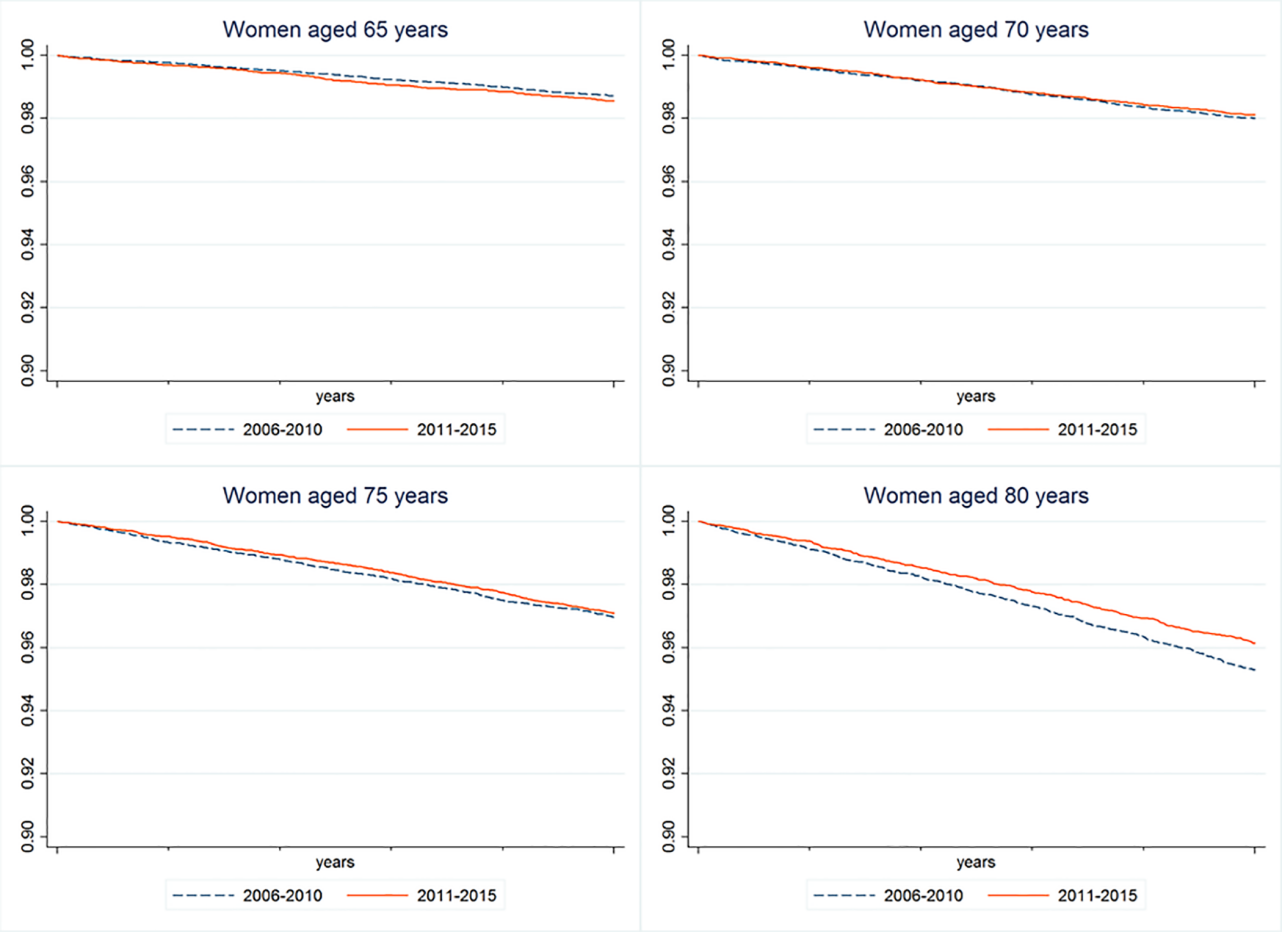
Survival curves of MI-onsets in women of different age groups (65–69 yrs., 70–74 yrs., 75–79 and 80–84 yrs.) for two periods (2006–2010 and 2011–2015).

In **women** (Fig 2) the comparisons of the survival curves of the first four age intervals (60-64/ 65-69/ 70–74 years) were not statistically significant. Differences between time periods emerged only for women aged 80 to 84 years (chi^2^ = 9.79; p = 0.001), indicating decreasing MI-rates in 2011 to 2015. This finding was not reproduced for the highest age group (85–89 years; chi^2^ = 2.1; p = 0.15).

Taken together, it can be concluded that the MI-related findings presented in the preceding two lines of analysis are mainly due to the age groups 70 to 79 years in men and 80 to 84 years in women.

## Discussion

Our study was conducted to examine morbidity compression with myocardial infarction as particular application. A population-based dataset was available that permitted to consider morbidity and mortality within the same database by examining two variants of morbidity compression: Changes of rates and change of age at occurrence.

Three findings have to be mentioned: It turned out that in women and in men MI-rates were decreasing over the whole observation period, but only in men this was also observed for mortality. MI-rates were decreasing at a faster pace than mortality rates, thus pointing towards compression of morbidity. Further analyses revealed that the developments of rates were mainly due to changes in the age groups 70 to 79 years in men, while in women this occurred beyond the age of 80. Decreasing trends of MI-incidences were also reported for Australia [59], Sweden [60], for the USA [38] and for Germany [39], but the relationship with mortality rates had rarely been explored. The second finding refers to age at onset and at death that were going up only in men, while this was absent in women. An earlier study with male military personnel from the US reported changes into the same direction [40], but again there is a lack of findings combining MI-morbidity and mortality. Our findings are also demonstrating that increasing onset age and decreasing rates as variations of morbidity compression are not necessarily intertwined, instead they may vary independently. The third finding refers to the marked gender differences that have to be interpreted against the backdrop of higher female longevity and higher mean age at MI-onset. Our findings may also be interpreted as part of a gender convergence driven by the development in men.

Irrespective of considerations on healthy longevity, in our study MI-rates have decreased and morbidity compression has occurred in men and in women. Fries assumed prevention and health-related behaviors to be the main driving forces [8, 61]. Unfortunately, our database does not include behavioral data that can be linked with our claims dataset. For this reason, explanations have to be developed with reference to other studies. Smoking was demonstrated to make a substantial contribution to the development of cardiovascular diseases [62], and tobacco consumption was reported to do more damage to the health of women than of men [63]. In high-income countries the proportions of smokers have decreased in the last decades, and smoking rates of women were approaching those of men [27, 64]. According to nationwide German surveys the proportion of smokers between 25 and 69 years dropped from 39.5% in 1990 to 34.9% in 2012. Among females, only minor changes of rates occurred as 26% were smoking in 1990 and 28.4% in 2012 after a peak of 32% in 2003 [63]. Exercise is another health-related behavior associated with the risk of MI. Changes of exercise habits of the German population were documented for the time period 1994 to 2011. In 30 to 64-year old individuals the proportion of women and men who have taken exercise was constantly increasing, and this also applies to the whole range of physical activity [65]. Data on the consumption of nutrition in middle and old aged women and men were only available on a cross-sectional basis [66]. Besides lifestyles also social factors may explain variations of morbidity and mortality, and well-established health-related influences are unemployment and the structure of work. Although health-related consequences of unemployment and adverse working conditions [67–69] have generated a large body of research, no longitudinal studies are available that can be made useable for explaining morbidity compression.

If compression is depicted in terms of decreasing morbidity rates, implications for general health have to be considered. The first one might be an improvement of health status, because cardiovascular diseases are affecting the health of populations, and reducing these burdens might directly contribute to morbidity compression. The second one might be a postponement of morbidity into higher age groups where other types of diseases and impairments may occur more frequently. This refers to illnesses such as stroke or clusters of health impairments that might best be characterized as multimorbidity [70]. Both interpretations are in accordance with morbidity compression, but the decision between them is open and subject to further investigation.

Our analyses were also pertaining to mortality at the level of a complete population by assuming that MI-onsets are part of general morbidity that in turn contributes to the risk of death. As a criticism of our approach it may be argued that case-fatality (death after MI-onset) might be a better indicator than all-cause mortality, or age at death. However, it has to be kept in mind that shortened survival after MI might not be interpreted in terms of morbidity compression, but as failure of medical treatment. Fries pointed out that morbidity compression is a population concept and that morbidity and mortality do not need to be observed in the same individuals [16, 61, 71].

For every observation year, the annual mean increment of age at MI-onset or at death in men was around one month. As mentioned earlier, this may be due to the social structure of our insurance population that does not fully correspond to the population of the Land of Lower Saxony, or also to Germany as a whole [45]. Some other limitations of our data have to be mentioned. Studying morbidity compression in terms of myocardial infarction is an important, but only a first step towards exploring the empirical content of Fries' hypothesis. Our dataset does not permit explanations as no data on living conditions and health-related behaviors were available, but it has the advantage of large case numbers at population level what permitted to consider a specific disease with a clear diagnosis. Another shortcoming of our database is the lack of privately insured subjects, i.e. civil-service personnel, officials and the upper decile of the income distribution [47]. In 2014 11% of the German population was falling into these categories. Studies on social inequalities in health have demonstrated inverse relationships between socioeconomic position and disease risks [72]. Against the backdrop of the literature on health inequalities it can be assumed that morbidity compression may develop differently depending on the socio-economic groups considered. Thus, our findings may underestimate the degree of compression within the whole population of Germany.

## Conclusions

Our study found evidence in favor of morbidity compression. In terms of MI-onsets, compression of morbidity has occurred in men and in women, and this was due to changes in specific age groups. In men the developments of morbidity and mortality rates combined with the development of age and age at onset were pointing towards morbidity compression. In contrast, the development in women was less straightforward. The substantial drop of MI-rates along constant mortality rates was indicating morbidity compression while age at onset and age at death remained unchanged. Our findings have shown that changes of rates and age at death may vary independently, thus emphasizing that compression is a multi-faceted phenomenon.
